# A Non-inflammatory Role for Microglia in Autism Spectrum Disorders

**DOI:** 10.3389/fneur.2016.00009

**Published:** 2016-02-01

**Authors:** Catherine A. Edmonson, Mark N. Ziats, Owen M. Rennert

**Affiliations:** ^1^University of Florida College of Medicine, Gainesville, FL, USA; ^2^National Institute of Child Health and Human Development, National Institutes of Health, Bethesda, MD, USA; ^3^Medical Scientist Training Program, Baylor College of Medicine, Houston, TX, USA

**Keywords:** autism spectrum disorder, neurodevelopment, microglia, glia, neurodevelopmental disorders

## Abstract

Autism spectrum disorder (ASD) is a neurodevelopmental disorder characterized by deficits in social interaction, difficulties with language, and repetitive/restricted behaviors. The etiology of ASD is still largely unclear, but immune dysfunction and abnormalities in synaptogenesis have repeatedly been implicated as contributing to the disease phenotype. However, an understanding of how and if these two processes are related has not firmly been established. As non-inflammatory roles of microglia become increasingly recognized as critical to normal neurodevelopment, it is important to consider how dysfunction in these processes might explain the seemingly disparate findings of immune dysfunction and aberrant synaptogenesis seen in ASD. In this review, we highlight research demonstrating the importance of microglia to the development of normal neural networks, review recent studies demonstrating abnormal microglia in autism, and discuss how the relationship between these processes may contribute to the development of autism and other neurodevelopmental disorders at the cellular level.

## Introduction

**Figure 1:**
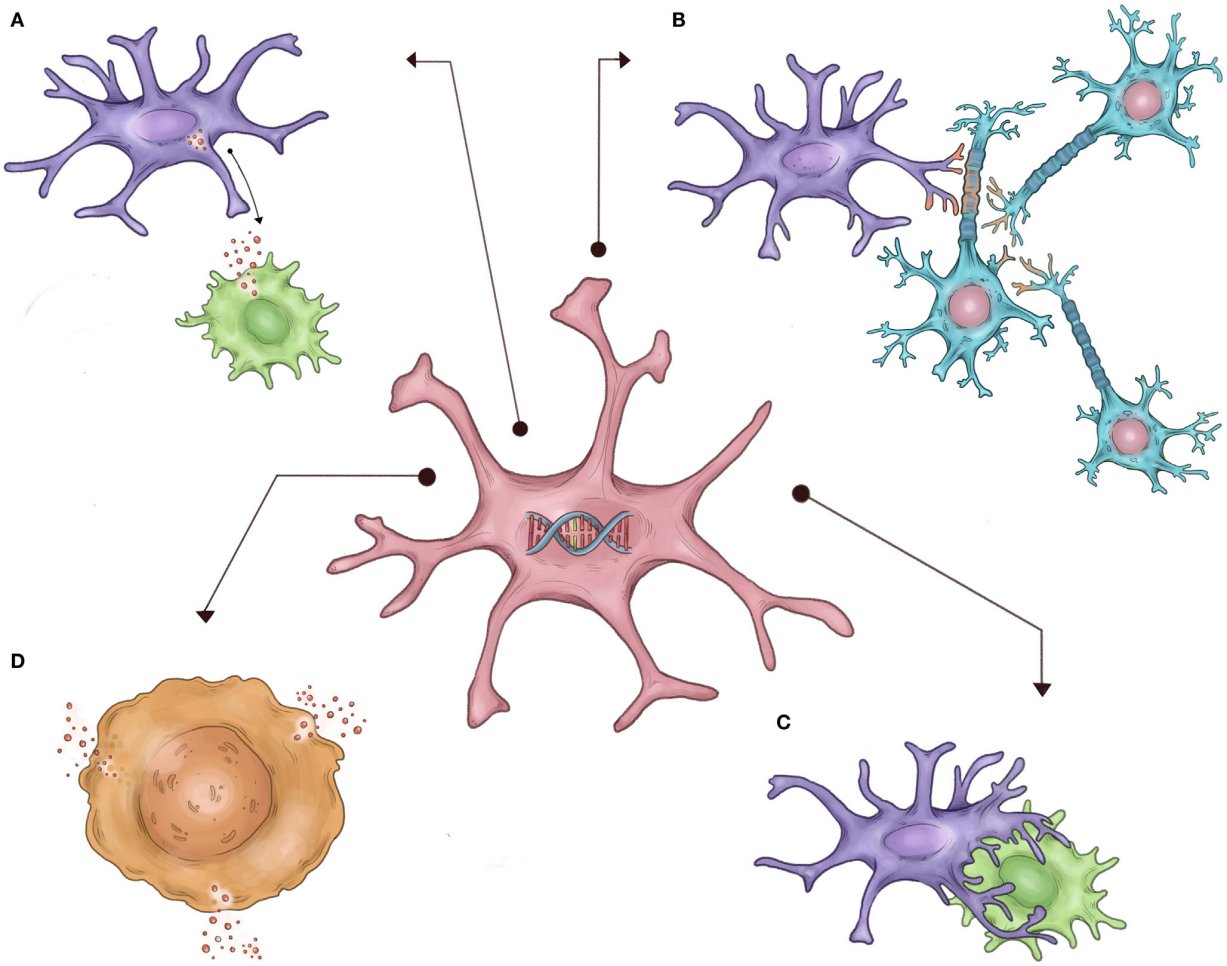
**Schematic representation of hypothesized non-immune microglial contributions to ASD, as described in the text**. It is proposed that inherited defects in the microglial genome/epigenome in autistic patients (center) result in abnormal or exaggerated execution of normal developmental microglial functions, such as **(A)** abnormal secretion of trophic factors necessary for normal neuronal growth, **(B)** incorrect synaptic pruning, **(C)** failure of appropriate apoptosis of neurons, and **(D)** exaggerated activation and cytokine secretion.

Autism spectrum disorder (ASD) is a clinical neurodevelopmental syndrome characterized by abnormalities in social interaction and language, and restrictive/repetitive patterns of interest (1). Despite the large and seemingly increasing prevalence of ASD (2), the biological mechanisms underlying the broad ASD phenotype remain unclear. However, strong evidence has recently implicated both immune pathways and independently, abnormalities in neural synaptogenesis, as potentially underlying the clinical ASD phenotype. However, it is unclear how these two seemingly distinct processes are related to the risk for the development of autism. A large body of research has suggested a link between inflammation in the CNS and resultant destruction of neural networks as one potential link between these two processes (3). However, microglia – the resident immune cells of the CNS – are increasingly implicated in *normal* neurogenesis and the formation of neural networks in the unaffected developing brain (4–6). Furthermore, microglia have also been repeatedly shown to be abnormal in postmortem autistic brain tissue and cellular/animal models of ASD (7–10). Therefore, it is equally likely that inherent abnormalities in microglia that are required to properly shape developing neural networks may link these two seemingly disparate processes separate from microglia’s traditional role as immune cells (Figure 1). In this review, we highlight research demonstrating the importance of microglia to the development of normal neural networks, review recent studies demonstrating abnormal immune signaling and microglia in ASD, and discuss how the relationship between these two emerging areas of neurodevelopment research may at least partially contribute to the development of ASDs at the cellular level.

## Discussion

### Microglia in Normal Neurodevelopment

The microglial cells of the CNS derive from myeloid progenitor cells in the yolk sac in early gestation, and travel to the brain to establish residency by early embryonic development (11). Traditionally, cellular neuroscience research has considered glial cells as predominately supportive to neurons during normal neurogenesis. In particular, microglia were traditionally viewed very narrowly as only becoming activated in response to pathological insults, similar to their peripheral tissue counterparts, and thus contributing little to normal neurodevelopmental processes (12–14). However, *in vivo* imaging techniques have repeatedly demonstrated that microglia are highly active during normal brain development and that they interact directly with neurons during critical period of neurogenesis (4, 5). Moreover, activity-dependent synapse and dendritic spine remodeling have been shown to be modulated in part by microglia in the mouse motor cortex and visual cortex (6). These and similar findings have helped to shift the view of normal cellular neurodevelopment from a primarily neuroncentric process to one that acknowledges the interactions between neurons and glia in properly wiring the brain’s circuitry under normal physiological conditions.

Recent studies support the idea that microglial cells play a central role in early neurodevelopment; however, the specific mechanisms of how microglia contribute and what effect abnormalities in their contribution may have is only beginning to be understood. Some have suggested that microglia may be responsible for releasing trophic factors essential for early neuronal growth. This idea is supported by studies demonstrating that cultures of neuronal progenitor cells lacking microglia have decreased proliferation as compared to cultures containing microglia, and notably, this phenotype was rescued when microglia were readded to the culture medium (15). Additionally, it has been shown that inactivating microglia in the developing mouse cortex on postnatal day 5 resulted in increased neuronal apoptosis in layer 5 of the cerebral cortex (16). These studies imply that absence of trophic or other factors released by microglia have the potential to significantly alter the number of neurons within a specific brain region and therefore change the normal developmental trajectory of that region.

In addition to providing trophic support to developing neurons, it has also been shown that microglial cells play a central role in the normal postnatal apoptosis and phagocytosis of neurons and their connections, which are naturally over-produced and then “pruned” away based on experience-dependent usage (17). Specifically, numerous studies over the last decade have shown that microglia are essential in both initiating cell death and phagocytizing dying neurons in the developing retina, spinal cord, cerebellum, hippocampus, and cerebral cortex (12, 18). It is not completely understood how exactly microglia carry out this process of cell death; however, it is thought to involve the release of nerve growth factors, the production of superoxide ions, and/or the increased expression of microglial surface proteins CD11b and DAP12, which initiate signal transduction leading to apoptosis (19). Complement activation has also been implicated in the mechanism of synaptic pruning in microglia (20–22). This idea is supported by data showing that disruption of CR3/C3 signaling leads to increases in synapse numbers and connectivity (23). Interestingly, we previously found differential expression of *DAP12* in the prefrontal cortex (PFC) of postmortem brain tissue from children with ASD as compared to controls (24). Additionally, it is known that mutations in *DAP12* lead to Nasu–Hakola disease (OMIM# 221770), a rare autosomal recessive disorder characterized by bone abnormalities and adult-onset neuropsychiatric features, such as social disinhibition, distractibility, and lack of appropriate emotionality (25).

Apart from directly impacting the number of neurons in the developing brain, microglial cells have been shown to be involved in more nuanced aspects of synaptogenesis, such as in controlling the number of synapses, and in regulating synapse maturation and function (26–28). Synapse pruning is thought to be regulated by both spontaneous- and experience-driven processes, and recent studies have shown that microglia may be involved in both of these mechanisms. It has been shown that microglial cells contact dendritic spines, presynaptic terminals, and synaptic clefts during a critical period in the development of the mouse visual cortex, and additionally that the size of dendritic spines change after contact with microglia (29). Perhaps the most substantial mechanistic linkage of microglia to normal neurodevelopment and behavior was a study that assessed for both cellular and behavioral outcomes of mice lacking the Fractalkine receptor CX3CR1 that is expressed exclusively on microglia in the CNS (26). This study showed that *CX3CR1* knockout mice had delayed synaptic pruning, resulting in excessive and electrophysiologically immature synapses. Moreover, related studies have even suggested that the behavioral phenotype of these mice is altered. For instance, it was demonstrated that mice lacking CX3CR1 had decreased functional brain connectivity, deficits in synaptic pruning, and behavioral changes associated with the autism phenotype, such as deficits in social interaction and increased repetitive behaviors (30).

### Microglia in ASD

Although microglia’s role in normal neurodevelopment is beginning to be well-recognized, parallel research into the role of microglia in autism and other neurodevelopmental disorders apart from their classic inflammatory function is only in its infancy. However, there is a large body of literature assessing aberrant immune function in peripheral tissue of children with ASD. For instance, numerous studies have suggested the innate immune response is globally abnormal in autistic patients (31–34). Specifically, peripheral blood monocytes have been shown to differ significantly in autistic patients. For instance, they secrete a cytokine pattern upon stimulation that is altered and more responsive to certain TLR ligands (35). Autistic patients also have higher plasma levels of factors involved with normal macrophage/monocyte activation, such as macrophage inhibitory factor and neopterin, and some studies have shown increased absolute monocytes on standard complete blood count in children with ASD as compared to controls (36, 37). Additionally, chemokines were shown to be increased in the plasma of children with ASD compared to age-matched typically developing controls and children with developmental disabilities other than ASD, and this increased chemokine production was associated with higher aberrant behavior scores and more impaired developmental and adaptive function (33). However, only more recently have rigorous assessment of microglia in postmortem brain tissue from patients with ASD been undertaken.

In 2005, Vargas and colleagues demonstrated that postmortem brain tissue from patients with autism exhibits an increased microglial density in gray matter and an activated microglial morphology; additionally, altered cytokine profiles were found in both ASD postmortem brain tissue and cerebrospinal fluid (7). Since this study, multiple other groups have reported increased microglial cell density in postmortem autism brains (8, 10), and we also reported increased numbers of microglial cell-specific surface markers in postmortem autism PFC as compared to control brains (24). Notably, the cellular volume of the activated microglia is two to four times the volume of quiescent microglial cells, and thus the increase in cell density may be partly related to increases in the number of activated microglia not overall microglial cell number alone (8). Additionally, it has been proposed that an increase in activated microglia may explain the increase in head circumference and brain volume observed in young children with autism (38), although this remains controversial as does the finding of increased head circumference in ASD in general (39).

In addition to cell-level studies of microglia in autistic postmortem brain tissue, a number of whole-genome expression studies have investigated RNA expression patterns in ASD postmortem brain tissue as compared to neurotypical controls (40–42). A recurrent finding in these studies is alterations in functional gene ontology pathways related to the immune response. For instance, dysregulated levels of immune system-related genes have been demonstrated in multiple independent studies that have assessed many different brain regions (41–46). While these studies show that genes related to “immune” functions are aberrantly expressed in postmortem brain tissue from patients with ASD, they do not clarify whether underlying genetic variants cause this expression profile or if this pattern represents an epigenetic response to either endogenous or exogenous brain insults. Future work assessing for enrichment of genetic variants in genes exclusively expressed in microglia among patients with ASD would help answer this question and could be undertaken readily. Studies of epigenetic changes in individual cell types from postmortem autistic brain tissue may yield additional critical insight into the relative contribution of environmental factors on this repeatedly demonstrated gene expression profile.

While postmortem brain studies have begun identifying abnormal microglia in ASD, by their nature, they are inherently limited in attempting to ascertain if abnormal microglia are a reaction to, or the cause of, abnormal neurodevelopment. However, recent studies in rodent models of autism have begun to explore this question in more mechanistic detail. For instance, it was found that microglia from *MeCP2* null mice, a model of Rett syndrome, produced a conditioned media that damaged synaptic connectivity *via* a glutamate-excitotoxicity mechanism (9). Importantly, much of the phenotype associated with this disorder was reversed after transplanting wild-type microglia with a functional *MeCP2* gene, suggesting microglia play a key role in the pathogenesis of this disease. Moreover, it was recently demonstrated that the Rett phenotype may be partly related to *MeCP2* direct modulation of microglial inflammatory gene transcription (47). Similarly, a large body of work has demonstrated that maternal inflammation during gestation may result in phenotypes similar to ASD in both primates and rodents (48–50). This important work begins to bridge the gap in understanding between inherited risk for ASD and environmental exposures; yet, a mechanistic understanding of this work that relates to known postmortem findings in humans with ASD is not yet clear nor does it fully encompass the known genetic risk for ASD. Our hypothesis presented here would be further supported by these studies, in that the inherited risk for ASD could result in “primed” abnormal microglial cells that in individuals exposed to maternal inflammation or other factors results in an exaggerated/abnormal microglial response that perturbs normal neural network development (48).

Much work remains to definitively link abnormal microglia with the broad autistic phenotype; however, the evidence presented provides strong support to the notion that microglial cells may be able to reconcile the two most consistent gene expression and cellular findings in autism – changes in synaptogenesis and immune pathways. This hypothesis is particularly exciting as inherent “hypomorphic” abnormalities in microglia at the genetic or epigenetic level and their resultant impaired functioning in neurodevelopment may help to explain the substantial contribution to autism risk that is thought to arise from interactions among intrinsic and environmental factors (51).

## Conclusion

In summary, mounting evidence suggests that normal neurodevelopment represents a complex interplay between microglial cells and synaptic wiring. Furthermore, non-inflammatory roles for microglia in normal neurodevelopment are becoming increasingly identified. In parallel, abnormal immune signaling and microglial function are consistently demonstrated in postmortem tissue from autistic individuals as well as in mouse models of ASD. Previous research into altered immune function in autism has focused on the notion that microglia may cause destruction of already developed neural networks, perhaps in response to external insults, through their traditional inflammatory role. However, as their critical non-inflammatory role in normal neurodevelopmental becomes increasingly recognized, it is equally likely that this is what may be perturbed in ASD, yet was traditionally considered to be “immune dysregulation.” Future research should attempt to integrate the genetic and epigenetic etiology, neuronal synaptic disconnectivity, and abnormal microglia/immune findings in autism in an attempt to determine the precise role non-immune functions of microglia may play in the pathogenesis of ASD and other neurodevelopmental disorders.

## Author Contributions

CAE and MNZ contributed equally to the conception, writing, and editing of the manuscript. OMR was involved in the conception and editing of the manuscript.

## Conflict of Interest Statement

The authors declare that the research was conducted in the absence of any commercial or financial relationships that could be construed as a potential conflict of interest.

## References

[1] American Psychiatric Association. Diagnostic and Statistical Manual of Mental Disorders: DSM-5. 5th ed Washington, DC: American Psychiatric Association (2013).

[2] CDC. Prevalence of autism spectrum disorder among children aged 8 years—autism and developmental disabilities monitoring network, 11 sites, United States, 2010. MMWR (2014) 63(Suppl. 2):1–21. 24670961

[3] Mead J Ashwood P. Evidence supporting an altered immune response in ASD. Immunol Lett (2015) 163:49–55. 10.1016/j.imlet.2014.11.006 25448709

[4] Davalos D Grutzendler J Yang G Kim JV Zuo Y Jung S ATP mediates rapid microglial response to local brain injury in vivo. Nat Neurosci (2005) 8:752–8. 10.1038/nn1472 15895084

[5] Nimmerjahn A Kirchhoff F Helmchen F. Resting microglial cells are highly dynamic surveillants of brain parenchyma in vivo. Science (2005) 308:1314–8. 10.1126/science.1110647 15831717

[6] Parkhurst CN Yang G Ninan I Savas JN Yates JR Lafaille JJ Microglia promote learning-dependent synapse formation through brain-derived neurotrophic factor. Cell (2013) 155:1596–609. 10.1016/j.cell.2013.11.030 24360280 PMC4033691

[7] Vargas DL Nascimbene C Krishnan C Zimmerman AW Pardo CA. Neuroglial activation and neuroinflammation in the brain of patients with autism. Ann Neurol (2005) 57:67–81. 10.1002/ana.20315 15546155

[8] Morgan JT Chana G Pardo CA Achim C Semendeferi K Buckwalter J Microglial activation and increased microglial density observed in the dorsolateral prefrontal cortex in autism. Biol Psychiatry (2010) 68:368–76. 10.1016/j.biopsych.2010.05.024 20674603

[9] Derecki NC Cronk JC Lu Z Xu E Abbott SBG Guyenet PG Wild-type microglia arrest pathology in a mouse model of Rett syndrome. Nature (2012) 484:105–9. 10.1038/nature10907 22425995 PMC3321067

[10] Suzuki K Sugihara G Ouchi Y Nakamura K Futatsubashi M Takebayashi K Microglial activation in young adults with autism spectrum disorder. JAMA Psychiatry (2013) 70:49–58. 10.1001/jamapsychiatry.2013.272 23404112

[11] Ginhoux F Greter M Leboeuf M Nandi S See P Gokhan S Fate mapping analysis reveals that adult microglia derive from primitive macrophages. Science (2010) 330:841–5. 10.1126/science.1194637 20966214 PMC3719181

[12] Bessis A Béchade C Bernard D Roumier A. Microglial control of neuronal death and synaptic properties. Glia (2007) 55:233–8. 10.1002/glia.20459 17106878

[13] Pollard JW. Microglial physiology: unique stimuli, specialized responses. Annu Rev Immunol (2009) 27:119–45. 10.1146/annurev.immunol.021908.132528 19302036

[14] Harry GJ. Microglia during development and aging. Pharmacol Ther (2013) 139:313–26. 10.1016/j.pharmthera.2013.04.013 23644076 PMC3737416

[15] Antony JM Paquin A Nutt SL Kaplan DR Miller FD. Endogenous microglia regulate development of embryonic cortical precursor cells. J Neurosci Res (2011) 89:286–98. 10.1002/jnr.22533 21259316

[16] Ueno M Fujita Y Tanaka T Nakamura Y Kikuta J Ishii M Layer V cortical neurons require microglial support for survival during postnatal development. Nat Neurosci (2013) 16:543–51. 10.1038/nn.3358 23525041

[17] Shatz CJ Impulse activity and the patterning of connections during CNS development. Neuron (1990) 5:745–56. 10.1016/0896-6273(90)90333-B 2148486

[18] Sierra A Abiega O Shahraz A Neumann H. Janus-faced microglia: beneficial and detrimental consequences of microglial phagocytosis. Front Cell Neurosci (2013) 7:6. 10.3389/fncel.2013.00006 23386811 PMC3558702

[19] Bilimoria PM Stevens B Microglia function during brain development: new insights from animal models. Brain Res (2014) 1617:7–17. 10.1016/j.brainres.2014.11.032 25463024

[20] Stevens B Allen NJ Vazquez LE Howell GR Christopherson KS Nouri N The classical complement cascade mediates CNS synapse elimination. Cell (2007) 131:1164–78. 10.1016/j.cell.2007.10.036 18083105

[21] Schafer DP Stevens B. Synapse elimination during development and disease: immune molecules take centre stage. Biochem Soc Trans (2010) 38:476–81. 10.1042/BST0380476 20298206

[22] Stephan AH Barres BA Stevens B. The complement system: an unexpected role in synaptic pruning during development and disease. Annu Rev Neurosci (2012) 35:369–89. 10.1146/annurev-neuro-061010-113810 22715882

[23] Schafer DP Lehrman EK Kautzman AG Koyama R Mardinly AR Yamasaki R Microglia sculpt postnatal neural circuits in an activity and complement-dependent manner. Neuron (2012) 74:691–705. 10.1016/j.neuron.2012.03.026 22632727 PMC3528177

[24] Edmonson C Ziats MN Rennert OM. Altered glial marker expression in autistic post-mortem prefrontal cortex and cerebellum. Mol Autism (2014) 5:3. 10.1186/2040-2392-5-3 24410870 PMC3914711

[25] Paloneva J Manninen T Christman G Hovanes K Mandelin J Adolfsson R Mutations in two genes encoding different subunits of a receptor signaling complex result in an identical disease phenotype. Am J Hum Genet (2002) 71:656–62. 10.1086/342259 12080485 PMC379202

[26] Paolicelli RC Bolasco G Pagani F Maggi L Scianni M Panzanelli P Synaptic pruning by microglia is necessary for normal brain development. Science (2011) 333:1456–8. 10.1126/science.1202529 21778362

[27] Tremblay M-È Stevens B Sierra A Wake H Bessis A Nimmerjahn A. The role of microglia in the healthy brain. J Neurosci (2011) 31:16064–9. 10.1523/JNEUROSCI.4158-11.2011 22072657 PMC6633221

[28] Schafer DP Lehrman EK Stevens B The “quad-partite” synapse: microglia-synapse interactions in the developing and mature CNS. Glia (2013) 61:24–36. 10.1002/glia.22389 22829357 PMC4082974

[29] Tremblay M-È Lowery RL Majewska AK. Microglial interactions with synapses are modulated by visual experience. PLoS Biol (2010) 8:e1000527. 10.1371/journal.pbio.1000527 21072242 PMC2970556

[30] Zhan Y Paolicelli RC Sforazzini F Weinhard L Bolasco G Pagani F Deficient neuron-microglia signaling results in impaired functional brain connectivity and social behavior. Nat Neurosci (2014) 17:400–6. 10.1038/nn.3641 24487234

[31] Brynskikh A Warren T Zhu J Kipnis J. Adaptive immunity affects learning behavior in mice. Brain Behav Immun (2008) 22:861–9. 10.1016/j.bbi.2007.12.008 18249087

[32] Ashwood P Krakowiak P Hertz-Picciotto I Hansen R Pessah IN Van de Water J Associations of impaired behaviors with elevated plasma chemokines in autism spectrum disorders. J Neuroimmunol (2011) 232:196–9. 10.1016/j.jneuroim.2010.10.025 21095018 PMC3053074

[33] Ashwood P Krakowiak P Hertz-Picciotto I Hansen R Pessah I Van de Water J Elevated plasma cytokines in autism spectrum disorders provide evidence of immune dysfunction and are associated with impaired behavioral outcome. Brain Behav Immun (2011) 25:40–5. 10.1016/j.bbi.2010.08.003 20705131 PMC2991432

[34] Goines PE Croen LA Braunschweig D Yoshida CK Grether J Hansen R Increased midgestational IFN-γ, IL-4 and IL-5 in women bearing a child with autism: a case-control study. Mol Autism (2011) 2:13. 10.1186/2040-2392-2-13 21810230 PMC3170586

[35] Enstrom AM Onore CE Van de Water JA Ashwood P. Differential monocyte responses to TLR ligands in children with autism spectrum disorders. Brain Behav Immun (2010) 24:64–71. 10.1016/j.bbi.2009.08.001 19666104 PMC3014091

[36] Sweeten TL Posey DJ McDougle CJ. High blood monocyte counts and neopterin levels in children with autistic disorder. Am J Psychiatry (2003) 160:1691–3. 10.1176/appi.ajp.160.9.1691 12944347

[37] Schlegelmilch T Henke K Peri F. Microglia in the developing brain: from immunity to behaviour. Curr Opin Neurobiol (2011) 21:5–10. 10.1016/j.conb.2010.08.004 20817438

[38] Schumann CM Nordahl CW. Bridging the gap between MRI and postmortem research in autism. Brain Res (2011) 1380:175–86. 10.1016/j.brainres.2010.09.061 20869352 PMC3050078

[39] Amaral DG Schumann CM Nordahl CW. Neuroanatomy of autism. Trends Neurosci (2008) 31:137–45. 10.1016/j.tins.2007.12.005 18258309

[40] Purcell AE Jeon OH Zimmerman AW Blue ME Pevsner J. Postmortem brain abnormalities of the glutamate neurotransmitter system in autism. Neurology (2001) 57:1618–28. 10.1212/WNL.57.9.1618 11706102

[41] Garbett K Ebert PJ Mitchell A Lintas C Manzi B Mirnics K Immune transcriptome alterations in the temporal cortex of subjects with autism. Neurobiol Dis (2008) 30:303–11. 10.1016/j.nbd.2008.01.012 18378158 PMC2693090

[42] Voineagu I Wang X Johnston P Lowe JK Tian Y Horvath S Transcriptomic analysis of autistic brain reveals convergent molecular pathology. Nature (2011) 474:380–4. 10.1038/nature10110 21614001 PMC3607626

[43] Hu VW Frank BC Heine S Lee NH Quackenbush J. Gene expression profiling of lymphoblastoid cell lines from monozygotic twins discordant in severity of autism reveals differential regulation of neurologically relevant genes. BMC Genomics (2006) 7:118. 10.1186/1471-2164-7-33 16709250 PMC1525191

[44] Gregg JP Lit L Baron CA Hertz-Picciotto I Walker W Davis RA Gene expression changes in children with autism. Genomics (2008) 91:22–9. 10.1016/j.ygeno.2007.09.003 18006270

[45] Enstrom AM Lit L Onore CE Gregg JP Hansen RL Pessah IN Altered gene expression and function of peripheral blood natural killer cells in children with autism. Brain Behav Immun (2009) 23:124–33. 10.1016/j.bbi.2008.08.001 18762240 PMC2636576

[46] Lintas C Sacco R Persico AM. Genome-wide expression studies in autism spectrum disorder, Rett syndrome, and Down syndrome. Neurobiol Dis (2012) 45:57–68. 10.1016/j.nbd.2010.11.010 21130877

[47] Cronk JC Derecki NC Ji E Xu Y Lampano AE Smirnov I Methyl-CpG binding protein 2 regulates microglia and macrophage gene expression in response to inflammatory stimuli. Immunity (2015) 42:679–91. 10.1016/j.immuni.2015.03.013 25902482 PMC4407145

[48] Knuesel I Chicha L Britschgi M Schobel SA Bodmer M Hellings JA Maternal immune activation and abnormal brain development across CNS disorders. Nat Rev Neurol (2014) 10:643–60. 10.1038/nrneurol.2014.187 25311587

[49] Giovanoli S Weber-Stadlbauer U Schedlowski M Meyer U Engler H. Prenatal immune activation causes hippocampal synaptic deficits in the absence of overt microglia anomalies. Brain Behav Immun (2015) 15:30022–30022. 10.1016/j.bbi.2015.09.015 26408796

[50] Machado C Whitaker AM Smith SEP Patterson PH Bauman MD. Maternal immune activation in nonhuman primates alters social attention in juvenile offspring. Biol Psychiatry (2015) 77:823–32. 10.1016/j.biopsych.2014.07.035 25442006 PMC7010413

[51] Kim YS Leventhal BL. Genetic epidemiology and insights into interactive genetic and environmental effects in autism spectrum disorders. Biol Psychiatry (2015) 77:66–74. 10.1016/j.biopsych.2014.11.001 25483344 PMC4260177

